# Isolated Marginal Mandibular Nerve Injury Due to Prone Positioning During Cervical Spine Surgery: A Case Report

**DOI:** 10.7759/cureus.106144

**Published:** 2026-03-30

**Authors:** Tarsem Motten, Sabarathinam R Subramaniyam, Rashid Anjum, Sohail Sayyad, Abhinav Gupta

**Affiliations:** 1 Department of Orthopedics, All India Institute of Medical Sciences, Vijaypur, Jammu, Vijaypur, IND

**Keywords:** facial nerve palsy, marginal mandibular nerve, neuropraxia, posterior cervical spine surgery, prone positioning

## Abstract

Injury to the marginal mandibular nerve (MMN) as an isolated complication of posterior cervical spine surgery performed in the prone position is exceedingly rare, with only a handful of documented instances in indexed surgical literature. We present the case of a 60-year-old gentleman who developed unilateral lower facial deviation immediately following a seven-hour posterior cervical decompression and multilevel instrumented stabilization procedure. The deficit was confined entirely to the distribution of the MMN, with no involvement of other facial nerve branches or adjacent peripheral nerves. The clinical findings were most consistent with positional MMN neuropraxia, and the patient was managed conservatively with physiotherapy and ENT guidance, with early evidence of recovery before discharge. This report aims to draw the attention of spine surgeons to a complication that is probably underrecognized and underreported and to underline the anatomical and technical considerations that can reduce its occurrence.

## Introduction

The facial nerve, after leaving the stylomastoid foramen, fans out within the parotid gland into its five terminal branches: temporal, zygomatic, buccal, marginal mandibular, and cervical. Among these, the marginal mandibular nerve (MMN) is of particular clinical importance due to its superficial course and vulnerability to injury. It innervates the depressor anguli oris, depressor labii inferioris, and mentalis muscles, which contribute to lower lip movement and facial expression [1]. Even a temporary loss of MMN function causes a noticeable and functionally problematic asymmetry in the lower face. This includes an inability to depress the lower lip on the affected side, leading to asymmetry of the oral commissure, and, in some patients, trouble keeping saliva at the mouth’s angle. This asymmetry is instantly apparent to the patient and their family, imposing a considerable psychological load, even when the prognosis for recovery is favorable. In clinical practice, the MMN is most commonly injured during parotidectomy, submandibular gland excision, neck dissection, and facelift surgery [2]. This is explained by its anatomical course: the nerve runs in a superficial plane, lying deep to the platysma and superficial to the facial vessels [3]. Cadaveric studies demonstrate that in approximately 15-25% of individuals, the nerve descends below the inferior mandibular border before coursing superiorly, thereby increasing its susceptibility to surgical injury and external compression at this site [4].

However, isolated MMN injury due to intraoperative positioning, particularly during prone spinal surgery, is rare and likely underreported. Prone positioning is universally used for posterior spinal surgery because of the excellent access it affords to the posterior elements. Peripheral nerve injuries are a well-documented hazard of this position, with the brachial plexus, the ulnar nerve at the elbow, and the common peroneal nerve at the fibular head being the structures most commonly affected [5]. Facial nerve involvement is distinctly uncommon in this context, and isolated injury restricted to the MMN without any other branch of the facial nerve being affected is a rarer occurrence still. Peripheral nerve injuries related to intraoperative positioning are well recognized; however, isolated involvement of the MMN in prone spine surgery remains exceedingly rare and underreported.

We report such a case of isolated left MMN neuropraxia following a seven-hour posterior cervical decompression and instrumented fusion and discuss the probable mechanism, differential diagnosis, management, and preventive considerations.

## Case presentation

A 60-year-old gentleman, a retired schoolteacher, presented with a four-year history of pain and progressive weakness in both upper limbs, associated with sensory impairment. The deterioration had been gradual initially but had accelerated considerably over the preceding two years, to the point where routine daily activities were compromised. There was no history of diabetes mellitus, hypertension, or any other systemic condition that could independently predispose to peripheral neuropathy or obscure the neurological presentation. Neurological evaluation indicated bilateral upper limb weakness accompanied by diminished feeling within the C5-C8 dermatomal distribution (Table 1).

**Table 1:** Motor power assessment on presentation as per the modified Medical Research Council Scale Source: Compston (2010) [6]

Myotome	Right upper limb	Left upper limb
C5	4/5	2/5
C6	3/5	3/5
C7	4/5	2/5
C8	1/5	1/5

**Figure 1:**
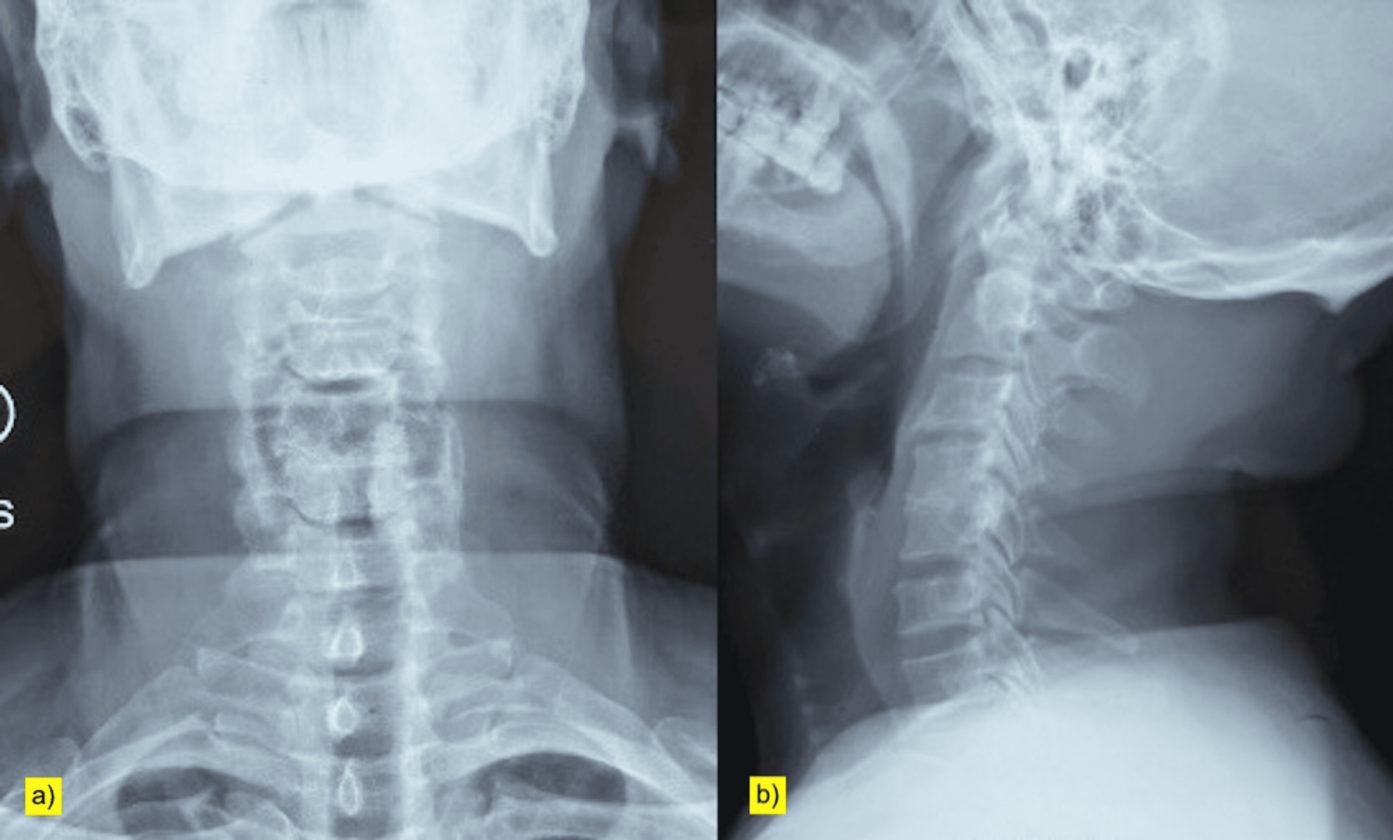
Plain radiographs of the cervical spine (a) Anteroposterior and (b) lateral views showing normal cervical alignment with no obvious bony abnormality.

**Figure 2:**
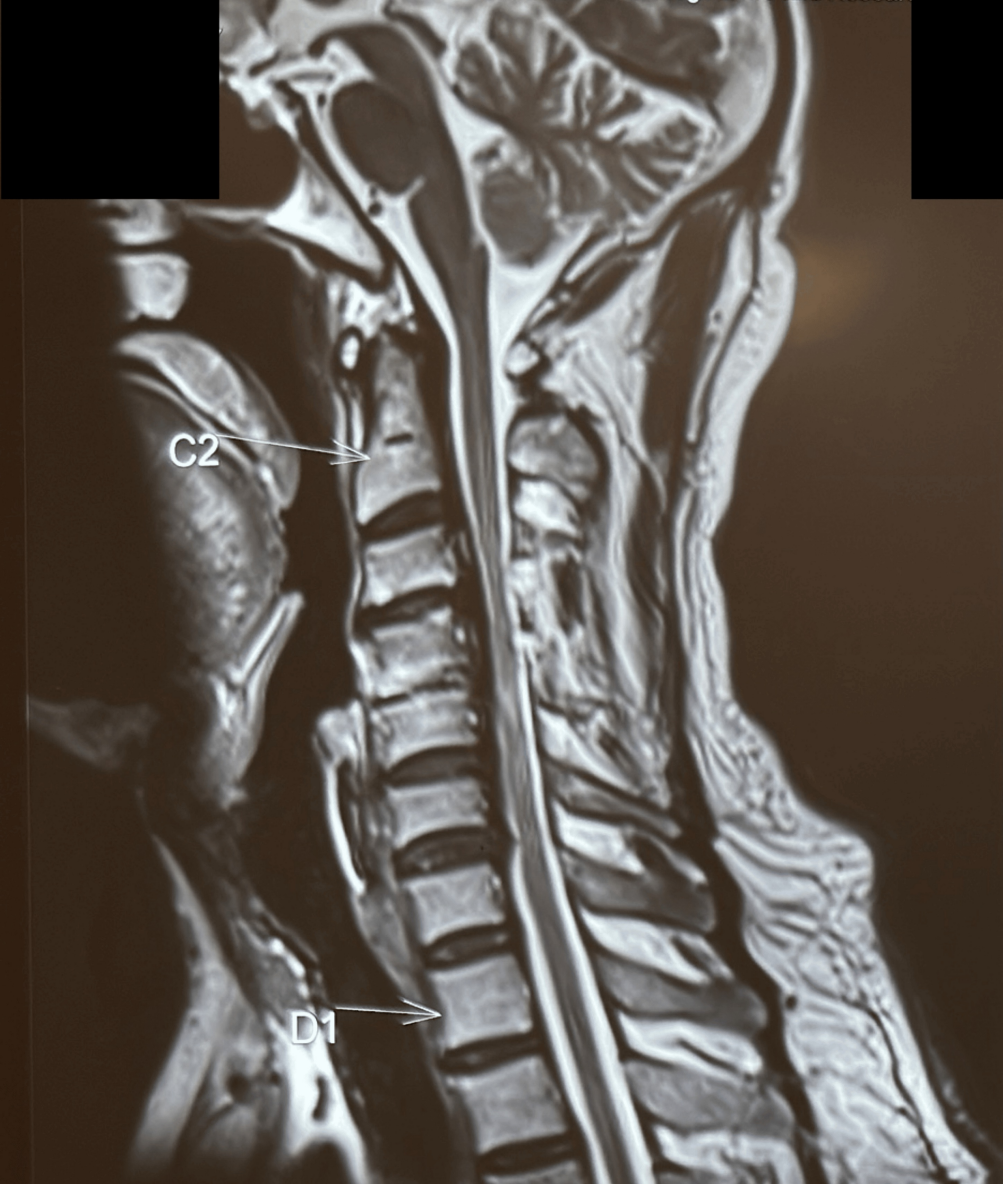
MRI of the cervical spine demonstrated significant multilevel stenosis with myelopathic signal changes within the cord, consistent with the clinical presentation

Radiographs of the cervical spine were obtained (Figure 1). MRI of the cervical spine demonstrated significant multilevel stenosis with myelopathic signal changes within the cord, consistent with the clinical presentation (Figure 2).

A supervised program of conservative management, cervical collar immobilization, analgesics, and physiotherapy was trialed for three months without meaningful benefit, following which surgical intervention was decided upon.

**Figure 3:**
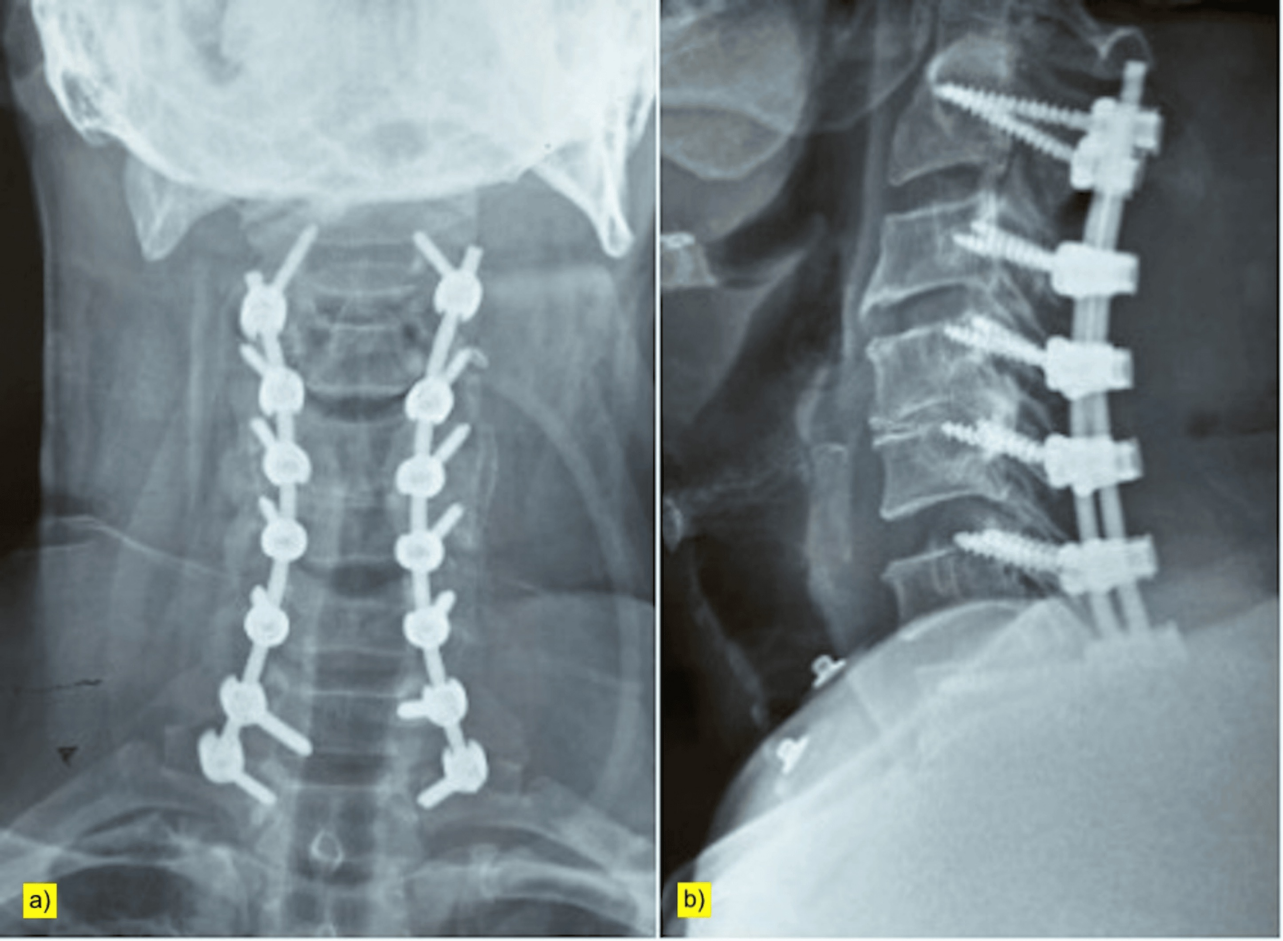
Postoperative cervical spine radiographs (a) Anteroposterior and (b) lateral views showing posterior screw-rod instrumentation from C2 to T1, with pedicle screws at C2, C7, and T1 and lateral mass screws from C3 to C6, following posterior cervical decompression.

The surgical procedure involved posterior cervical decompression and instrumented stabilization from C2 to T1. Pedicle screw fixation was placed at C2, C7, and T1; lateral mass screw fixation was performed at C3 through C6. Laminectomy was then carried out from C3 to C7, and foraminotomies were performed at C2 and bilaterally at C4 and C5 (Figure 3).

**Figure 4:**
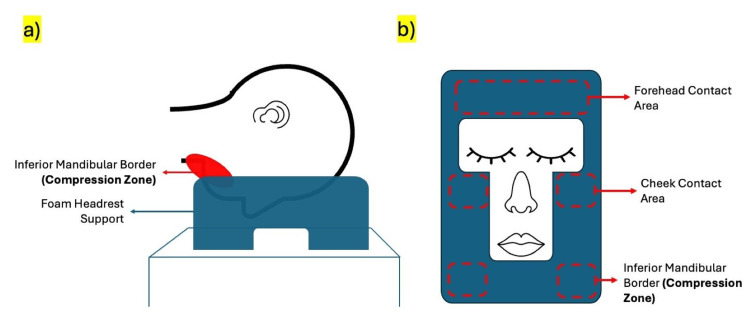
Prone positioning using a foam headrest showing facial contact zones and mandibular area (a) Lateral view demonstrating head and facial support during prone positioning. (b) Inferior view illustrating areas of contact at the forehead and cheeks, with relative sparing of the eyes and nose. The mandibular region lies at the interface between supported and unsupported zones and may be subjected to focal compression during prolonged procedures, placing the MMN at risk. MMN, marginal mandibular nerve Figure created using Microsoft PowerPoint (Microsoft Corporation, Redmond, WA, USA)

The procedure was performed under general anesthesia with the patient positioned prone using a foam headrest designed to support the forehead and allow free positioning of the face (Figure 4). Care was taken to ensure neutral head alignment and to avoid excessive pressure over facial structures. However, the mandibular region remains susceptible to indirect compression against the support surface, particularly during prolonged procedures.

**Figure 5:**
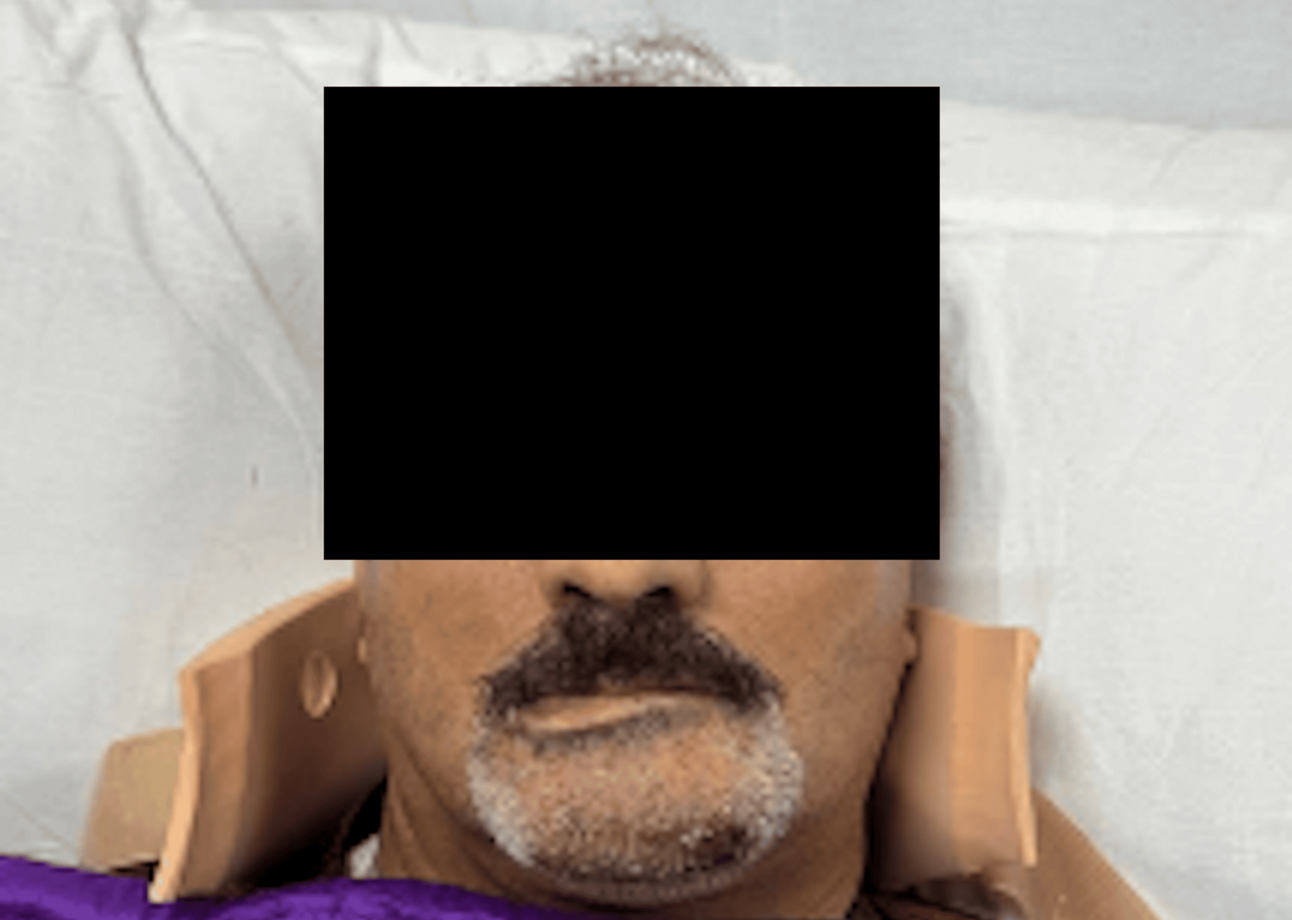
Post-op clinical image showing asymmetry of the lower lip, inability to depress the affected side, and preservation of upper facial movements

The operative duration was approximately seven hours. Immediately on reversal of anesthesia and extubation in the operating room, the patient was noted to have a deviation of the mouth towards the right side, with an inability to depress the left lower lip (Figure 5). He was otherwise alert and oriented, and the neurological deficits in the upper limbs were unchanged from the preoperative baseline. Crucially, there was no involvement of the left forehead, no weakness of orbicularis oculi, and eye closure and corneal sensation were entirely intact. Overall, the findings were consistent with isolated lower lip dysfunction with preserved upper facial movements, suggesting a peripheral lesion localized to the MMN of the facial nerve.

**Figure 6:**
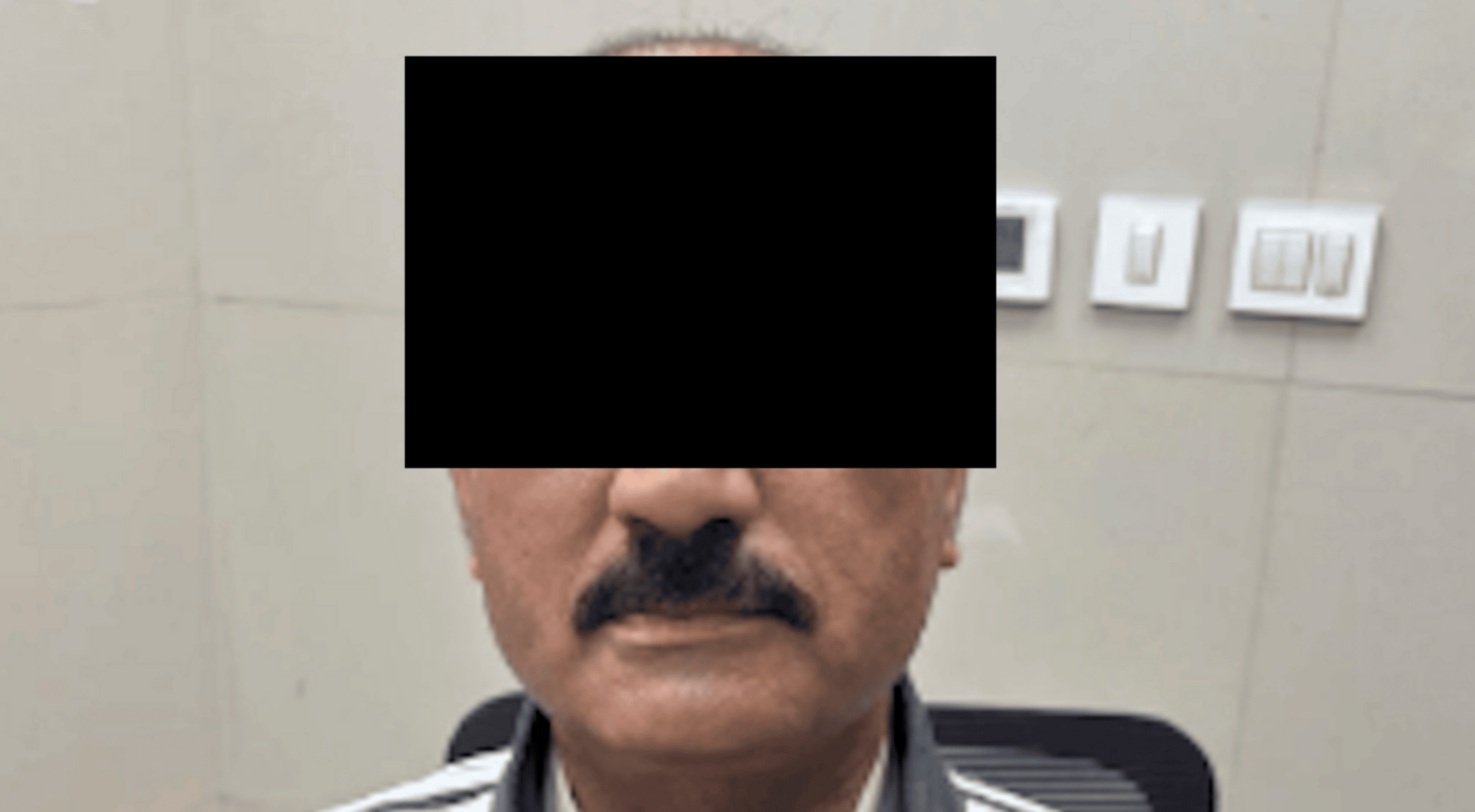
Clinical image of post-op one-month follow-up visit showing lower lip asymmetry with an inability to depress the left side, consistent with MMN involvement, with preserved upper facial movements MMN, marginal mandibular nerve

An ENT consultation was secured on the same day. CT scans of the brain and cervical spine were done to rule out any new central event, and they were normal. A clinical diagnosis of positional MMN neuropraxia was made. Electromyography and nerve conduction testing were scheduled for outpatient follow-up due to logistical challenges in the immediate postoperative period. A conservative care regimen was started, which included targeted physiotherapy for the lower face, neuromuscular retraining exercises, and a brief course of low-dose oral prednisolone with the ENT specialists. The patient claimed subjective improvement in lower lip mobility by the time of release on the fifth postoperative day, and clinical examination showed a mild but clear symmetry of the oral commissure (Figure 6). He was advised to continue his physiotherapy regimen and was scheduled for a combined spine and ENT review at six weeks.

## Discussion

The mechanism underlying positional MMN neuropraxia in the prone-positioned surgical patient is likely due to direct extrinsic compression. In this position, the face rests against a padded support such as a standard foam head ring, a Mayfield horseshoe, or a pinned skull clamp, and the mandibular region inevitably makes contact with or is in proximity to the supporting surface. When the MMN takes a course that dips below the inferior mandibular border, which occurs in a significant anatomical variant, the nerve lies at the very point of maximum contact between the mandible and any underlying surface [3]. The platysma, which is the only soft-tissue layer superficial to the nerve at this point, provides minimal protection against sustained low-grade pressure over a period of hours.

The injury produced under these circumstances is neuropraxia, the least severe category in Seddon’s classification, characterized by localized conduction block without axonal or endoneurial disruption [7]. The ischemic mechanism of neuropraxia, in which even modest sustained pressure reduces intraneural blood flow sufficiently to impair axonal conduction, is well established in experimental models [8]. Recovery is typically complete and occurs over days to weeks; the maximum period generally quoted is three months, and long-term persistent deficit after proven neuropraxia would be unusual. Peripheral nerve injuries associated with prone positioning have been studied extensively in anesthetic closed-claims databases and surgical outcome registries [5]. The brachial plexus is cited most frequently, followed by the ulnar nerve at the elbow [9] and the common peroneal nerve at the fibular neck [10]. Facial nerve involvement is rare in this setting, and an injury restricted purely to the MMN sparing all other branches of the facial nerve is a striking anatomical specificity that points directly to a focal compressive mechanism at the point where the nerve is most superficial and most exposed. The absence of forehead involvement, intact eye closure, and normal corneal sensation together effectively exclude both central facial palsy and a more proximal or diffuse facial nerve injury, making any parotid, stylomastoid, or intracranial pathology highly unlikely.

A structured consideration of differential diagnoses is essential in this setting. Central facial palsy was considered; however, the preservation of forehead movement, intact eye closure, and absence of contralateral neurological deficits made a supranuclear lesion unlikely. A more proximal facial nerve injury at the level of the stylomastoid foramen or within the parotid gland was also unlikely, given the isolated involvement of the MMN without impairment of other facial nerve territories. Perioperative factors such as endotracheal tube fixation or external compression from surgical equipment were considered; however, the focal distribution of weakness corresponding precisely to the MMN, along with the immediate postoperative onset following prolonged prone positioning, supported a localized compressive neuropraxia as the most likely mechanism.

The duration of the prone position is an important variable. The anesthetic literature demonstrates a roughly exponential relationship between the duration of sustained positional pressure and the probability of nerve ischemia sufficient to produce conduction block [7]. Seven hours is a substantial operative duration, and it is not surprising that a nerve as anatomically exposed as the MMN would be susceptible at this length of time even with standard padding precautions. Longer procedures, multilevel reconstructions, revision surgeries, and complex deformity corrections are therefore higher-risk situations that warrant specific attention to this possibility during preoperative positioning planning. Intraoperative neurophysiological monitoring has transformed the safety profile of complex spinal procedures [11]. However, standard somatosensory and motor evoked potential monitoring does not evaluate the facial nerve, and free-running electromyography of the facial musculature is not a part of the standard monitoring protocol for posterior cervical procedures. The question of whether targeted facial nerve electromyographic monitoring should be considered during particularly prolonged prone surgeries remains open and may warrant prospective study.

The management of positional MMN neuropraxia is overwhelmingly conservative. Facial physiotherapy targeting the depressor group of muscles, patient education regarding the expected natural history of recovery, and reassurance constitute the primary intervention. There is no randomized controlled evidence specifically addressing corticosteroid use in this setting, but the extrapolation from Bell’s palsy trials where early prednisolone therapy has demonstrated significant benefit in accelerating recovery provides a reasonable justification for its cautious use [12]. Surgical exploration of the nerve is not indicated unless there is electrophysiological evidence suggesting axonotmesis or neurotmesis or if spontaneous recovery fails to progress beyond the expected three-month window. Prevention is unquestionably preferable. At the practical level, this means the operating surgeon taking personal responsibility for verifying facial positioning before the drapes go up, rather than treating this as solely an anesthetic concern [13]. The mandibular region should be free of any direct hard-surface contact. Gel foam padding, which distributes pressure more evenly than firm foam, is preferable at all facial contact zones. In patients with a lean facial constitution, prominent mandibular anatomy, or previous surgery involving the parotid or submandibular region, any of which might alter the normal soft-tissue cushioning over the nerve, extra vigilance is warranted. Periodic intraoperative checks of head position during lengthy procedures should be standard practice.

Limitations

This paper pertains to a singular case, resulting in equally restricted conclusions. Electrophysiological evidence of the injury was not achieved during the acute period, and long-term follow-up data are lacking. These are recognized limitations intrinsic to the documentation of infrequent perioperative occurrences, underscoring the significance of multicenter registries and comprehensive case series in the investigation of unusual surgical consequences.

## Conclusions

Isolated MMN neuropraxia is an uncommon but genuinely occurring complication of prolonged prone positioning during posterior cervical spine surgery. The injury likely results from localized compressive ischemia of the nerve at the point where it crosses the inferior mandibular border in a superficial anatomical plane. When the impairment is found, the clinical diagnosis is easy to make, and the prognosis is good with conservative therapy.

Despite its rarity, this complication warrants attention. It is not on the standard mental checklist of positional complications that most spine surgeons have, and if a clinician is not ready for it, an unfamiliar finding right after surgery can make the patient worry and cause unnecessary diagnostic work. Future prospective registries of postoperative neurological problems in spine surgery ought to incorporate dedicated capture fields for facial nerve impairments, thus facilitating a more accurate determination of the true prevalence of this and other positional injuries.
